# Management and outcome of multifetal gestation in a 35-year-old woman with childhood-onset membranoproliferative glomerulonephritis type I

**DOI:** 10.1007/s13730-018-0357-7

**Published:** 2018-08-12

**Authors:** Osamu Motoyama, Ken Sakai, Kikuo Iitaka

**Affiliations:** 1 Department of Pediatrics, Toho University Medical Center, Sakura Hospital, 564-1 Shimoshizu, Sakura-shi, Chiba, 285-8741 Japan; 2 Department of Nephrology, Toho University Medical Center, Omori Hospital, Tokyo, Japan; 3 Hakuraku Renal Clinic, Kanagawa, Japan

**Keywords:** Assisted reproduction technology, Chronic kidney disease, Membranoproliferative glomerulonephritis type I, Multifetal pregnancy

## Abstract

A 35-year-old woman with membranoproliferative glomerulonephritis type I had quintuplet gestation after induced ovulation. Her serum creatinine level and estimated glomerular filtration rate were 0.86 mg/dL and 61.5 mL/min/1.73 m^2^ before pregnancy. Blood pressure was normal and urinary protein to creatinine ratio was 0.2 g/gCr. Prednisolone 10 mg on alternate-day administration was continued during pregnancy. At 10 weeks of gestation transvaginal selective embryo reduction was performed and five embryos were reduced to twins. Hypertension occurred at 20 weeks of gestation. She developed nephrotic syndrome and serum creatinine level increased to 1.29 mg/dL. Elective cesarean section was performed at 28 weeks of gestation and dichorionic diamniotic twins were born. After delivery blood pressure, serum creatinine level, estimated glomerular filtration rate and serum albumin level in their mother returned to baseline. Her twin infants were well at discharge from neonatal-intensive-care-unit. Incidence of multifetal pregnancies due to the improvement of assisted reproduction technologies and ovulation-inducing hormones has been increasing. Management for multifetal pregnancy in women with chronic kidney disease will be needed further.

## Introduction

Membranoproliferative glomerulonephritis (MPGN) type I is a chronic glomerulonephritis characterized by hypocomplementemia, mesangial proliferation and double contours glomerular capillaries on light microscopy, deposits of C3 along the capillary walls on immunofluorescent microscopy, and mesangial interposition and subendothelial electron dense deposits on electron microscopy. According to the earlier reports during 1980, poor outcome of pregnancy in women with MPGN type I was suggested [1, 2]. In these reports the effects of hypertension, renal dysfunction or heavy proteinuria during pregnancy was not discussed. MPGN associated with hepatitis C virus infection was not excluded from idiopathic MPGN until 1993 [3]. Progress of obstetrical management and perinatal care improved prognosis of preterm and low birth weight infant [4, 5], even in women with chronic kidney disease (CKD) [6]. In recent years successful pregnancy in women with MPGN type I has been reported [3, 7, 8]. Incidence of multifetal pregnancies due to the improvement of assisted reproduction technologies and ovulation-inducing hormones has been increasing [9]. Multifetal pregnancies in women with CKD may increase. Clinical course of multifetal pregnancy in a woman with childhood-onset MPGN type I has not been reported to our knowledge.

## Case report

**Fig. 1:**
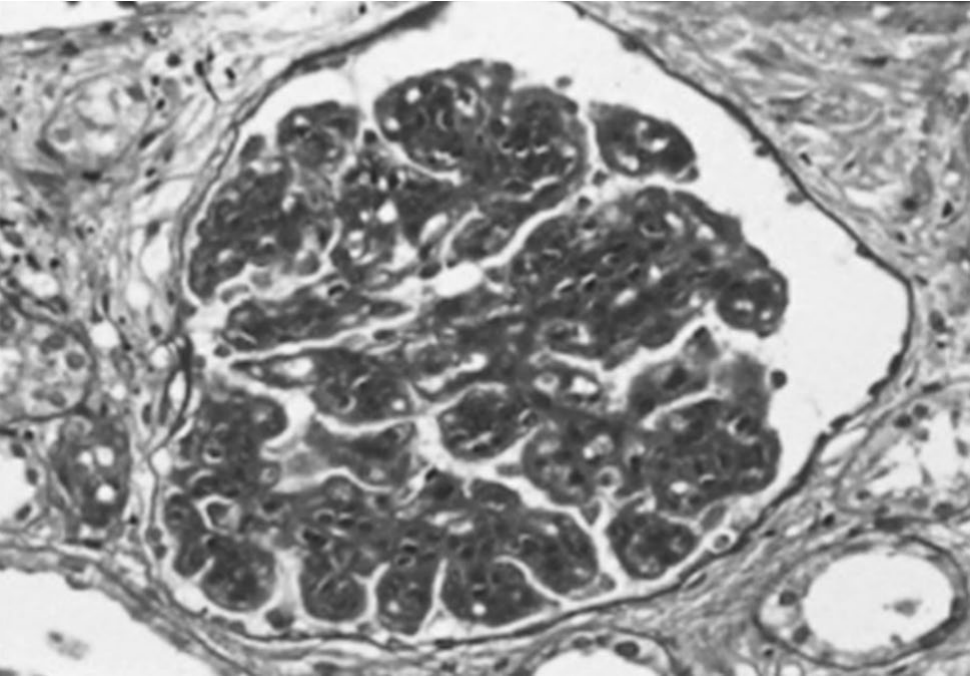
Light micrograph of first renal biopsy performed at the age of 11 years old showing increase of mesangial cells and matrix with a lobular pattern of glomeruli and thick glomerular capillary walls with double contours. Periodic-acid-Schiff stain, ×400

**Fig. 2:**
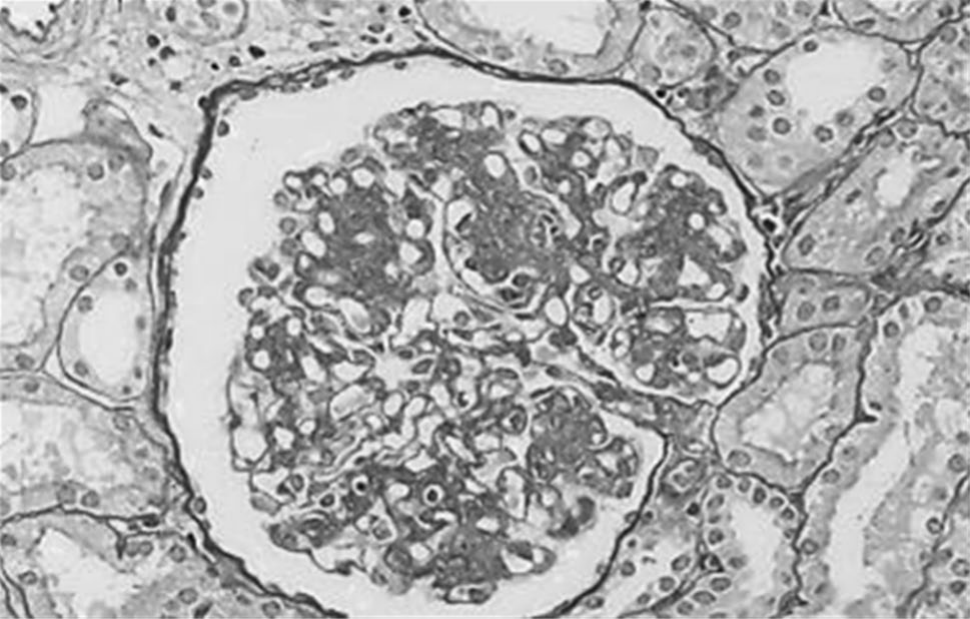
Light micrograph of second renal biopsy performed at the age of 14 years old showing decreased mesangial proliferation and capillary wall thickness compared to first renal biopsy findings. Periodic-acid-Schiff stain, ×400

Microscopic hematuria and proteinuria were detected by school urinary screening when she was 10 years old. Edema and hypertension were not noted. Hypocomplementemia was detected and the serum levels of complement hemolytic activity (CH50), C3 and C4 were 24 U/mL (normal range 28–48 U/mL), 21 mg/dL (normal range 64–166 mg/dL) and 19 mg/dL (normal range 15–38 mg/dL), respectively. Microscopic hematuria, proteinuria and hypocomplementemia continued and she developed nephrotic syndrome (serum albumin level 2.5 g/dL and urinary protein excretion 3.4 g/day) with normal blood pressure and renal function. The first renal biopsy was performed at 11 years of age and 30 glomeruli were obtained. Increase of mesangial cells and matrix with a lobular pattern of glomeruli and thick glomerular capillary walls with double contours were observed on light microscopic examination (Fig. 1). Subendothelial and mesangial deposits were observed on masson trichrome stain. There was no tubulointerstitial change. Lumpy C3 deposits along glomerular capillaries were demonstrated by immune-enzyme method (PAP method). There was weak staining of IgA, IgG and IgM along glomerular capillaries. Glomeruli were not included in the specimen for electron microscopic studies. There were no clinical signs or symptoms of systemic lupus erythematosus, thrombotic microangiopathy and malignancies. Anti-DNA antibody, hepatitis B virus antigen, hepatitis C virus antibody and cryoglobulins were negative. MPGN type I was diagnosed. Prednisolone (PSL) was started with 60 mg (2 mg/kg/day) for 4 weeks and gradually reduced to 15 mg on alternate days over 1 year period. Urinary protein excretion decreased to 2+ by dipstick and hypoalbuminemia and hypercholesterolemia improved after the treatment with PSL and dipyridamole [10]. Microscopic hematuria, proteinuria and hypocomplementemia continued at 14 years of age and the follow-up renal biopsy was performed; 18 glomeruli were obtained (Fig. 2). Mesangial proliferation and capillary wall thickness were decreased. Lobulation of glomeruli was not noted. Fibro-cellular crescents in 3 glomeruli and a sclerosed glomerulus were noted. There was no tubulointerstitial change. Immunofluorescent micrograph showed dominant C3 deposition along the capillary walls, weak IgG staining and traces of IgA and IgM in the mesangium and along the capillary walls. Findings on electron microscopy were not available. Pathological findings were thought to be improved and PSL was reduced to 10 mg on alternate days. At 16 years of age proteinuria increased to 3.4 g/day and she became nephrotic again. Serum creatinine level was normal (0.45 mg/dL). Intravenous methylprednisolone 500 mg per day for 3 days was administrated followed by oral PSL 10 mg on alternate days. Angiotensin-converting enzyme inhibitor for renoprotection was added. Hypocomplementemia improved (C3 > 64 mg/dL) at 19 years of age. At 25 years microscopic hematuria disappeared and hypoalbuminemia improved (serum albumin level > 2.5 g/dL). Serum creatinine was 0.82 mg/dL, estimated glomerular filtration rate (eGFR) was 71.6 mL/min/1.73 m^2^, and proteinuria was 3.7 g/day. During 26 and 32 years, her serum creatinine was between 0.85 and 1.06 mg/dL, eGFR was between 51.7 and 67.2 mL/min/1.73 m^2^, and morning spot urine protein/creatinine ratio was between 0.2 and 0.7 g/gCr. Blood pressure was normal and treatment with PSL 10 mg on alternate days was continued.

She wished to have a child even after the risks of maternal and neonatal complications associated with pregnancy, including preeclampsia, CKD development and preterm delivery, were explained to the patient and her husband. She had two gestations at 32 and 33 years of age and both resulted in spontaneous abortion at 9 weeks of gestation. She received ovulation induction with human menopausal gonadotropin and human chorionic gonadotropin at another Obstetrics and Gynecology Clinic and had quintuplet gestation. At 10 weeks of gestation transvaginal selective embryo reduction procedure was performed and five embryos were reduced to twins. After the procedure, she was referred to the Department of Obstetrics and Gynecology of Omori Hospital. Her body height, weight and body mass index before pregnancy were 154 cm, 55 kg and 23.2 kg/m^2^, respectively. After angiotensin-converting enzyme was discontinued at each gestation, her blood pressure was normal. At 20 weeks of the third gestation, hypertension occurred and administration of methyldopa was started. From 24 weeks she was admitted to the maternal-fetal-intensive-care-unit and anti-hypertensive drug was changed to nifedipine. Hypertension continued. Urinary protein excretion increased to 6.7 g/day and nephrotic syndrome developed. Serum creatinine level increased to 1.29 mg/dL and eGFR decreased to 39.1 mL/min/1.73 m^2^. Elective cesarean section was performed at 28 weeks of gestation. Twin babies were born. Male baby was 1235 g of weight, 34.5 cm of height and 29.4 cm of head circumstance at birth and was appropriate for date. His Apgar score was 6 at 5 min after birth. Female baby was 883 g of weight (below the 10th percentile for the Japanese population), 33.2 cm of height (above the 10th percentile) and 24.8 cm of head circumstance (above the 10th percentile) at birth. The asymmetrical type intrauterine growth restriction due to placental insufficiency and superimposed preeclampsia was diagnosed. Her Apgar score was 5 at 5 min after birth. The twins were dichorionic diamniotic and discordant. Pathology of placenta showed infarct in some focal regions without signs of chorioamnionitis. Both babies were treated with surfactant administration and mechanical ventilation for respiratory distress syndrome. By the neonatal screening tests, the female baby was diagnosed as having hypothyroidism at 1 month of age (free triiodothyronine 1.91 pg/mL; normal range 2.26–4.15 pg/mL, free thyroxine 0.52 ng/dL; normal range 1.01–1.67 ng/dL, thyroid stimulating hormone 150 µIU/mL; normal range 0.32–4.12 µIU/mL) and was treated with levothyroxine. Urinary iodine concentration of the baby was high (760 µg/L; normal range 121–271 µg/L). The mother had undergone hysterosalpingography after a second abortion at 34 years of age. Excessive maternal iodine intake due to iodinated contrast medium administration may cause hypothyroidism of the baby [11]. At 3 months of age, she received laser photocoagulation for retinopathy due to prematurity. Any other congenital anomaly was not noted in both babies. They were discharged from neonatal-intensive-care-unit on day 74 and 114, respectively. A month after delivery, blood pressure, serum creatinine, eGFR and serum albumin of the mother returned to baseline levels. Nifedipine was changed to angiotensin-converting enzyme. Six months later, proteinuria decreased to 2+ (1.1 g/gCr). Clinical findings of the patient are shown in Table 1.

**Table 1:** Clinical course of a woman with membranoproliferative glomerulonephritis type I

	At first renal biopsy	At second renal biopsy	Before first pregnancy	Before second pregnancy	Before third pregnancy	At cesarean section	1 month after delivery
Age (years)	11	14	31	32	34	35	35
Blood pressure (mmHg)	104/50	120/66	114/86	128/80	110/70	170/105	124/80
Serum creatinine (mg/dL)	0.37	0.44	0.95	0.89	0.86	1.29	0.89
eGFR (mL/min/1.73 m^2^)	134.3	119.7	56.6	60.2	61.5	39.1	58.7
Serum albumin (g/dL)	2.5	3.9	3.8	3.5	3.9	2.4	3.6
CH50 (U/mL)	24	41	42	40	41	53	–
C3 (mg/dL)	21	55	85	83	85	116	–
C4 (mg/dL)	19	24	20	19	21	28	–
Hematuria^a^	3+	1+	–	–	–	–	–
Proteinuria^a^	3+	2+	2+	2+	1+	3+	3+
(g/day or g/gCr)^b^	3.4 g/day	1.5 g/day	0.5 g/gCr	0.7 g/gCr	0.2 g/gCr	6.7 g/day	5.6 g/gCr
Prednisolone	60 mg/day	15 mg/ADT	10 mg/ADT	10 mg/ADT	10 mg/ADT	10 mg/ADT	10 mg/ADT

*eGFR* estimated glomerular filtration rate, *CH50* complement hemolytic activity, *normal CH50* 28–48 U/mL, *normal C3* 64–166 mg/dL, *normal C4* 15–38 mg/dL, *ADT* alternate-day administration

^a^Grade of hematuria and proteinuria detected by dipstick

^b^Urinary protein excretion in 24 h (g/day) or morning spot urine protein-to-creatinine ratio (g/gCr)

## Discussion

The effects of pregnancy on preexisting CKD depend on the severity of renal impairment and the presence of hypertension and heavy proteinuria. The prognosis of pregnancy in women with serum creatinine level of < 1.4 mg/dL, normal blood pressure and no heavy proteinuria is generally considered to be good. The rate of successful pregnancy diminishes with increasing severity of CKD [6]. Neonatal mortality of low birth weight infants (< 2500 g) in Japan decreased from 8.8% in 1980 to 0.8% in 2010. More than 90% of infants born at 28 weeks or later survive now [3, 4]. Due to the improvement of obstetrical and perinatal care, 99% of 504 pregnancies in women with CKD stages 1–5 resulted in live births during 2000–2013. But complication rates of pregnancy remain high. Elevated blood pressure, increased proteinuria and decreased renal function during pregnancy in most women with eGFR ≧ 60 mL/min/1.73 m^2^ (CKD stages 1 and 2) recovers within 6 weeks after the delivery. Renal dysfunction remained after delivery in a half of women with eGFR < 45 mL/min/1.73 m^2^ (CKD stage 3b). About one-third in women with eGFR < 30 mL/min/1.73 m^2^ (CKD stages 4 and 5) required renal replacement therapy within a year after delivery [6].

Incidence of multifetal pregnancies has been increasing, using assisted reproduction technologies and ovulation-inducing hormones. In general population, outcomes of multifetal pregnancy become worse with increased number of fetuses. Gestational hypertension occurred in 7% of the mothers with singleton, 13% with twins and 20% with triplets [12]. Cesarean section was performed in about a half of twin gestations and in 90% of triplet gestations during preterm period [13, 14]. The incidence of complications and outcomes in singleton and multifetal pregnant CKD women in two reports by Piccoli et al. [15, 16] are showed in Table 2. New-onset hypertension during singleton pregnancy occurred in 9% of women with CKD stages 1 and 2 (eGFR ≧ 60 mL/min/1.73 m^2^) and 48% of women with CKD stages 3–5 (eGFR < 60 mL/min/1.73 m^2^). New onset or doubling of proteinuria during singleton pregnancy was observed in 24% of women with CKD stages 1 and 2 and in 83% of women with CKD stages 3–5 [15]. Among 17 multifetal (15 twins and 2 triplets) pregnancies, 4 of 9 patients (44%) with normal blood pressure before pregnancy became hypertensive and new-onset proteinuria or doubling of proteinuria occurred in 6 of 17 patients (35%) [16]. Incidence of cesarean section and preterm delivery was high in singleton pregnancy, compared with those in general population and was over 90% in twin pregnancies. In both healthy and CKD women, singleton pregnancy was safer than multifetal pregnancy. But after the trial of induced ovulation, multifetal pregnancies are reduced to twins to increase the chances of delivering at least one viable fetus in many cases [17, 18].

**Table 2:** Reported complications and outcomes of singleton and multifetal pregnancy in women with chronic kidney disease

	Number of patients	New-onset hypertension during pregnancy (%)	New-onset or doubling of proteinuria during pregnancy (%)	Cesarean section (%)	Preterm delivery (%)	Neonatal death (%)
Singleton pregnancy [15]						
CKD stages 1–2^a^	457	9	24	52	29	0
CKD stages 3–5^b^	47	48	83	77	81	2
Multifetal pregnancy [16]						
CKD stages 1–2^a^	17^c^	44	35	100	94	7

*CKD* chronic kidney disease

^a^Estimated glomerular filtration rate ≧ 60 mL/min/1.73 m^2^

^b^Estimated glomerular filtration rate < 60 mL/min/1.73 m^2^

^c^15 twin and 2 triplet pregnancies

In this paper, quintuplet gestation in a woman with MPGN type I resulted in live twin births after superimposed preeclampsia. Despite fetal reduction, preeclampsia occurred. But close follow-up by obstetricians and cesarean section at an appropriate time resulted in the successful delivery of preterm twin infants. Although hypertension, growth failure of the placenta due to PSL, and intrauterine growth restriction due to hypoproteinemia may be complicated in some cases, most women with CKD seem to have live birth recently [6]. Among 17 multifetal pregnancies reported by Piccoli et al. [16], six women had delivered the babies using assisted reproductive technologies. Incidence of multifetal pregnancies using assisted reproduction technologies in CKD women seems to be increasing. Nephrologist must know the risks of multifetal pregnancy in CKD women of childbearing age. There is no protocol about the management for multifetal pregnancy in CKD women. The management should be determined individually. The decision should depend on progression of CKD and maternal age. Although increased incidence of congenital abnormalities in children born to women with CKD is not reported, long term effects on mother and infant is not clear [6]. Small for gestational age babies and preterm babies may have effects on increased risks for neurological deficits and development of diabetes, metabolic syndrome, cardiovascular diseases and CKD carried into adulthood.
